# Emergency and sequalae management of traumatic dental injuries: a systematic survey of clinical practice guidelines

**DOI:** 10.1186/s12903-023-03409-w

**Published:** 2023-09-30

**Authors:** Carlos Zaror, Andrea Seiffert, Naira Figueiredo Deana, Gerardo Espinoza-Espinoza, Claudia Atala-Acevedo, Rodrigo Diaz, Alonso Carrasco-Labra

**Affiliations:** 1 Department of Pediatric Dentistry and Orthodontics, Faculty of Dentistry, Universidad de La Frontera, Temuco, Chile; 2 Center for Research in Epidemiology, Economics and Oral Public Health (CIEESPO), Faculty of Dentistry, Universidad de La Frontera, Temuco, Chile; 3 Dental School, Faculty of Dentistry, Universidad de La Frontera, Temuco, Chile; 4 Department of Public Health, Faculty of Medicine, Universidad de La Frontera, Temuco, Chile; 5 Melbourne Dental School, University of Melbourne, Melbourne, Australia; 6 Programa de Magíster en Odontología, Facultad de Odontología, Universidad de La Frontera., Temuco, Chile; 7 Center for Integrative Global Oral Health, School of Dental Medicine, University of Pennsylvania, Philadelphia, PA USA

**Keywords:** AGREE II, Clinical practice guidelines, Systematic review, Traumatic dental injuries

## Abstract

**Background:**

The prevalence and consequences of traumatic dental injuries (TDI) make them a public health problem. Trustworthy TDI clinical practice guidelines (CPGs) assist clinicians in determining a diagnosis and guide them to the most appropriate therapy. The aim of this systematic survey was to identify and evaluate the quality of CPGs for the diagnosis, emergency management, and follow-up of TDIs.

**Materials and methods:**

A systematic search was carried out in MEDLINE, EMBASE, Epistemonikos, Trip database, CPG websites, and dental societies to identify documents providing recommendations for the emergency and sequelae management of TDIs. Reviewers assessed the included guidelines independently and in duplicate, using the AGREE II instrument. ANOVA or Student’s t-tests were used to determine the attributes of CPGs associated with the total score in AGREE II.

**Results:**

Ten CPGs published between 2010 and 2020 were included, mostly from Europe (n = 6). The overall agreement between reviewers was very good (0.94; 95%CI 0.91–0.97). The mean scores (the higher the score, the better the domain assessment) per domain were as follows: Scope and purpose 78.0 ± 18.9%; stakeholder involvement 46.9 ± 29.6%; rigour of development 41.8 ± 26.7%; clarity of presentation 75.8 ± 17.6%; applicability 15.3 ± 18.8%; and editorial independence 41.7 ± 41.7%. The overall mean rate was 4 ± 1.3 out of a maximum score of 7. Two guidelines were recommended by the reviewers for use in practice and rated as high quality. CPGs developed by government organizations showed a significantly higher overall score.

**Conclusions:**

The overall quality of CPGs on TDI was suboptimal. CPG developers should synthesize the evidence and formulate recommendations using high-quality methodologies and standards in a structured, transparent, and explicit way.

**Supplementary Information:**

The online version contains supplementary material available at 10.1186/s12903-023-03409-w.

## Introduction

A traumatic dental injury (TDI) is an impact injury that affects the tooth and its supporting structures [1]. TDIs are a serious public health problem due to their prevalence and their consequences for the quality of life of the affected patients [2]. The estimated prevalence of TDIs worldwide is 22.7% in primary teeth and 15.2% in permanent teeth, with an estimated global incidence rate of 2.82 (number of events per 100 persons per year) [3]. The study by Petti et al. (2018) on the global burden of TDIs shows that more than one billion people have had at least one TDI; if ranked as an acute/chronic disease or injury, TDI would rank as the 5th most prevalent condition worldwide [3].

Proper diagnosis of TDIs, together with treatment planning and follow-up, are fundamental for ensuring a favorable outcome and prognosis [4]. Nevertheless, this task is not easy to achieve because of the complexity of diagnosing TDIs and the multiple treatment options available. A recent systematic review showed insufficient knowledge of TDI prevention and emergency management by dental professionals worldwide [5]. This lack of expertise induces a significant variability in the management of TDIs, directly impacting the patient’s oral health and quality of life [6], along with high costs for health systems [7, 8].

One way to help clinicians to make a proper diagnosis, guide them to the most appropriate therapy and reduce clinical variability is through clinical practice guidelines (CPGs). CPGs are developed by a guideline panel, drawing up evidence-based recommendations to help health professionals, patients, and caregivers to make an appropriate decision in specific clinical circumstances [9].

Evidence shows that CPGs across dental specialties tend to be assessed as low quality, primarily associated with a lack of methodological rigour of development [10, 11] and problems in applicability [12, 13], making their implementation unreliable and their use difficult for patients, clinicians, and policy-makers. Poor quality CPGs may negatively influence patient care or have debatable applicability [14, 15].

There is no systematic quality assessment of CPGs for TDIs; therefore, little is known about their quality, potential impact, and applicability. The aim of this study was to identify and evaluate the quality of CPGs for the diagnosis, emergency management and follow-up of TDIs.

## Materials and methods

We carried out a systematic quality evaluation of CPGs for TDIs using the AGREE II tool and following a methodology published previously [10, 13]. We used the SPIDER framework to define our research question [16]: Sample – general population (children and adults); Phenomenon of Interest – recommendations for the emergency management or treatment of the consequences of TDIs; Design – clinical practice guideline; Evaluation –guideline quality; Research type – qualitative studies. We published the protocol in the Open Science Framework [17].

### Eligibility criteria

We included documents published in English, German, Portuguese, and Spanish that were self-declared as a guideline or provided recommendations for the emergency management or treatment of the consequences of TDIs. We only included the most recent version of the CPGs identified. We excluded CPGs that only provide recommendations for maxillo-facial trauma unrelated to TDIs, documents that lack recommendations, and discontinued CPGs.

### Information sources

We conducted a systematic search in the MEDLINE, EMBASE, Epistemonikos and Trip databases up to May 22, 2021. Guideline developers’ websites, repositories, Health Ministries and international dental scientific societies were also screened. This search was updated in May, 2022. We did not restrict the search by date or language. Details of the search strategy can be found in the supplementary data (Appendix S1).

### Selection of the guidelines

The titles, abstracts, and full texts were reviewed independently by two researchers (R.D., A.S.) in a 3-step process using Rayyan® software (www.rayyan.ai). Any discrepancies were resolved by a third reviewer (C.Z.).

### Data Collection process

Two reviewers (A.S., C.A., G.E. or N.F.D.) independently extracted the following characteristics of each CPG included: author, year, title, country, organization, language, scope (emergency or treatment), target population, method used for the quality assessment, and methodology by which the recommendations were developed.

### Critical assessment of CPGs

Two reviewers (C.A., C.Z., G.E., or N.F.D.) worked independently to rate the quality of each guideline with the AGREE II instrument. AGREE II comprises 23 items and six domains: (1) Scope and purpose; (2) Stakeholder involvement; (3) Rigour of development; (4) Clarity of presentation; (5) Applicability; and (6) Editorial independence. Each item is rated on a Likert scale ranging from 1 (lowest quality) to 7 (highest quality) points. AGREE II includes two overall quality ratings for each guideline: (i) an overall score of 1 to 7; and (ii) a reviewer recommendation classing it as “recommended”, “recommended with modifications”, or “not recommended” [18].

### Statistical analysis

The total AGREE II score was determined by totaling the scores of all items in each domain and then scaling the total score as a percentage of the highest possible score for the domain [18]. Discrepancies between reviewers that exceeded 3 points, or standard deviation (SD) in any item equal to or greater than 1.5 SD, were reassessed [10, 13]. The standardized score was calculated for each domain (range 0 to 100%) [18].

CPGs with a score of 60% or higher in at least three domains, including Rigour of development, were classified as high-quality [10, 11, 13].

Overall agreement among the reviewers was calculated using the intraclass coefficient with a 95% confidence interval (95%CI). Agreement of 0.01 to 0.20 was considered slight, 0.21 to 0.40 fair, 0.41 to 0.60 moderate, 0.61 to 0.80 substantial, and 0.81 to 1.00 very good [19].

ANOVA or Student’s t-tests were used to determine associations between the total score in AGREE II and the attributes of the CPGs, e.g. year of development (last five years or more), CPG development agencies (Government, Scientific societies, or hospitals), and region (Europe, America, Asia). Any significant ANOVA was checked by post-hoc tests (Tukey’s Honestly Significant Differences) to determine differences between groups.

Finally, we used the Pearson correlation coefficient to evaluate correlations between the AGREE II domain scores and the total score to establish which domains influenced the overall quality of the guidelines. Pearson’s correlation was interpreted as follows: r < 0.1 negligible, 0.1*–*0.39 weak, 0.4*–*0.69 moderate, 0.7–0.89 strong, and r > 0.9 very strong [20].

## Results

**Fig. 1:**
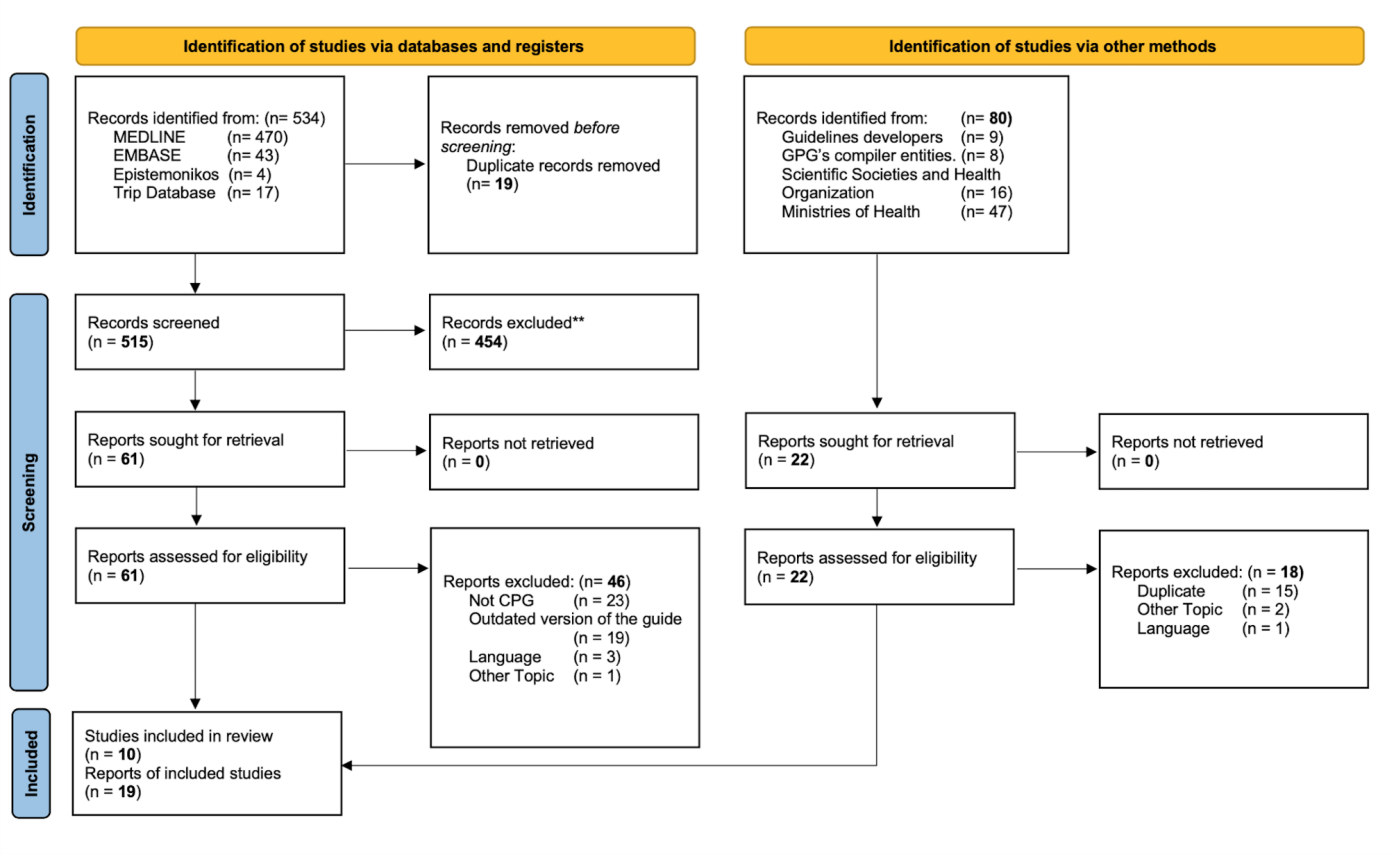
Flow diagram of the selection process

The selection flow chart is shown in Fig. 1. The systematic search retrieved 479 articles, and other sources identified 80 documents/articles. After excluding duplicates and studies that failed to meet the inclusion criteria, ten CPGs were included in the final analysis.

Table 1 lists the characteristics of the CPGs, which were all published between 2010 and 2022. Eight were in English: three from the UK; one each from Italy, Norway, Malaysia and the USA; and one global. Of the other two, one was in Spanish and the other in German. Six CPGs were focused on recommendations for managing all types of TDIs, two for avulsed teeth, one for intruded teeth and one for endodontic management of traumatized permanent teeth. The developers were scientific societies or dental colleges (n = 6), Ministries or government agencies (n = 3) and a Hospital (n = 1). Only two CPGs reported their funding source. Only three CPGs [4, 21–26] are recent updates from a previous version. Although most guidelines stated that they had conducted a systematic review (n = 8), only two assessed the risk of bias and certainty of evidence [27, 28]. Most of the guidelines that reported a methodology for the drafting of the recommendations did so through consensus [27].

**Table 1:** Characteristics of the guidelines included

Reference	Guideline	Year	Organization	Country	Scope (dentition)	Level of Development	Language	Funding Source	Systematic Review	Evidence Quality Assessment or Evidence Grading Methodology	Recommendation Development
[46–48]	Guía de Manejo de trauma dento alveolar	2011	Hospital de la Misericordia	Colombia	Emergency management of all types of TDI (Permanent)	Private Hospital	Spanish	Not reported	Yes	Not reported	Consensus
[49, 50]	Leitlinienreport zur S2k-Leitlinie Therapie des Dentalen Traumas bleibender Zähne	2022	German Society for Oral and Maxillofacial Surgery (DGMKG), German Society for Dental, Oral and Maxillofacial Medicine (DGZMK)	Germany	Emergency management of all types of TDI (Permanent)	Scientific Society	German	German Society for Dentistry, Oral and Maxillofacial Medicine, German Dental Association and National Association of Statutory Health Insurance Dentists	Yes	Not reported	Consensus
[23]	Italian guidelines for the prevention and management of dental trauma in children	2019	Italian Ministry of Health	Italy	TDI prevention and Emergency management (Primary and permanent)	Government	English	None	Yes	SNLG	Not reported
[4, 24–26]	International Association of Dental Traumatology guidelines for the management of traumatic dental injuries	2020	International Association of Dental Traumatology	International	Emergency management of all types of TDI (Primary and permanent)	Scientific Society	English	Not reported	Yes	Not reported	Consensus
[27]	Management of Avulsed Permanent Anterior Teeth	2019	Ministry of Health Malaysia	Malaysia	Emergency management of avulsion (Permanent)	Government	English	Ministry of Health Malaysia	Yes	GRADE	Consensus
[51]	European Society of Endodontology position statement: endodontic management of traumatized permanent teeth	2021	European Society of Endodontology	Norway	Endodontic management of TDI (Permanent)	Scientific Society	English	Not reported	Yes	Not reported	Consensus
[29]	UK National Clinical Guidelines in Paediatric Dentistry: treatment of traumatically intruded permanent incisor teeth in children	2010	British Society of Paediatric Dentistry	UK	Emergency management of intrusion (Permanent)	Scientific Society	English	Not reported	Not reported	SIGN	Not reported
[21, 22]	UK National Clinical Guidelines in Paediatric Dentistry Treatment of avulsed permanent teeth in children	2012	British Society of Paediatric Dentistry	UK	Emergency management of avulsion (Permanent)	Scientific Society	English	None	Yes	SIGN	Not reported
[28]	Management of Acute Dental Problems. Guidance for healthcare professionals	2013	Scottish Dental Clinical Effectiveness Programme (SDCEP)	UK	Emergency management of all types of TDI (Primary and permanent)	Government	English	Scottish Government and NHS Education for Scotland	Yes	AMSTAR	Consensus
[52]	The Recommended Guidelines of the American Association of Endodontists for the Treatment of Traumatic Dental Injuries	2014	American Association of Endodontics	USA	Emergency management of all types of TDI (Permanent)	Scientific Society	English	Not reported	Not reported	Not reported	Not reported

GRADE: Grading of Recommendations Assessment, Development and Evaluation; SIGN: Scottish, Intercollegiate Guidelines Network; SNLG: The Italian National Guideline System; AMSTAR: A Measurement Tool to Assess Systematic Reviews

### Guidelines assessment

The agreement between the reviewers was classed as very good (ICC = 0.94; 95%CI 0.91–0.97). Table 2 shows the standardized scores for each CPG by domain, and the overall recommendation. The only domains to score above 60% were Scope and purpose, and Clarity of presentation. The lowest score was the Applicability domain, with a mean of 15.3% ± 18.8.

**Table 2:** AGREE scores of CPGs by domain

Reference	Guideline	Scope and purpose	Stakeholder involvement	Rigour of development	Clarity of presentation	Applicability	Editorial independence	Overall rate	Overall Recommendation	Quality
[46–48]	Guía de Manejo de trauma dento alveolar	50%	22%	15%	36%	2%	0%	2	Not recommended	Low
[49, 50]	Leitlinienreport zur S2k-Leitlinie Therapie des Dentalen Traumas bleibender Zähne	97%	83%	51%	78%	10%	100%	4.5	Recommended with modifications	Low
[23]	Italian guidelines for the prevention and management of dental trauma in children	100%	67%	82%	83%	0%	96%	6	Recommended	High
[4, 24–26]	International Association of Dental Traumatology guidelines for the management of traumatic dental injuries	69%	17%	34%	86%	15%	25%	4.5	Recommended with modifications	Low
[27]	Management of Avulsed Permanent Anterior Teeth	92%	75%	52%	64%	56%	88%	5	Recommended with modifications	Low
[51]	European Society of Endodontology position statement: endodontic management of traumatized permanent teeth	75%	33%	33%	92%	0%	50%	3.5	Not recommended	Low
[29]	UK National Clinical Guidelines in Paediatric Dentistry: treatment of traumatically intruded permanent incisor teeth in children	58%	33%	20%	69%	13%	4%	2.5	Not recommended	Low
[21, 22]	UK National Guidelines in Paediatric Dentistry Treatment of avulsed permanent teeth in children	100%	50%	55%	94%	17%	0%	4.5	Recommended with modifications	Low
[28]	Management of Acute Dental Problems. Guidance for healthcare professionals	81%	86%	74%	67%	40%	54%	5	Recommended	High
[52]	The Recommended Guidelines of the American Association of Endodontists for the Treatment of Traumatic Dental Injuries	58%	3%	2%	89%	0%	0%	2.5	Not recommended	Low
	Mean	78.0%	46.9%	41.8%	75.8%	15.3%	41.7%	4.0		
	(SD)	18.9%	29.6%	25.7%	17.6%	18.8%	41.7.%	1.3		
	(Minimum–Maximum)	(50–100%)	(3–86%)	(2–82%)	(36–94%)	(0–56%)	(0-100%)	2–6		

### Scope and purpose

The mean score was 78.0% ± 18.9 (range 50–100%). Of the ten guidelines, seven scored above 60% in this domain, demonstrating that most of the guidelines defined well the target audience for whom the CPG was planned.

### Stakeholder involvement

Four CPGs scored above 60% in the Stakeholder involvement domain, while the mean score was 46.9% ± 29.6 (range 3–86%). The main limitations of this domain were the need for more detailed information about the group that developed the guideline (discipline, institution, description of role) and the failure to consider the preferences of target users.

### Rigour of development

For this domain, the mean score was 41.8% ± 25.7 (range 2–82%). Only two guidelines scored above 60%. Although most of the guidelines declared that they had conducted a systematic search of evidence, only two formally assessed the strengths and limitations of the supporting evidence [27, 28]. However, three guidelines graded the evidence of the studies included, in an effort to assess the quality of the supporting evidence [21–23, 29].

Scarce information was provided on the methods used to develop the recommendations. However, most CPGs used consensus as the method by which the panel members reached their decisions. Seven guidelines reported a direct link between the supporting evidence and the recommendations. Four CPGs reported information on external peer review prior to dissemination [21–23, 27, 28], and two reported appropriate information about the updating process [27, 28].

### Clarity of presentation

In this domain, the mean score was 75.8% ± 17.6% (range 36–94%). Only one CPG scored below 60% in this domain, indicating that the recommendations were clearly presented.

### Applicability

All the guidelines scored less than 60% in the Applicability domain. The mean score for this domain was 15.3% ± 18.8 (range 0–56%). The main limitations were that most of the CPGs did not discuss barriers to and facilitators of implementation, did not evaluate the implications of the use of resources, or did not present key review criteria for the purposes of monitoring and/or auditing [27, 28].

### Editorial independence

For this domain, the mean score was 41.7% ± 41.7 (range 0-100%). Seven CPGs scored below 60% and two of them scored 0.0%. Some CPGs did not fully describe their sources of funding and the possible influence of these on CPG development or failed to report the authors’ potential conflicts of interest.

### Overall assessment

Only two of the guidelines were classed by the reviewers as recommended, and four were recommended with modifications. After the assessment, two CPGs were classified as high quality (scored ≥ 60% in at least three domains, including Rigour of Development). The overall mean was 4 ± 1.3, the highest score awarded was 6, while the lowest was 2.

### AGREE II score and features of the Guidelines

The CPGs developed by governments showed a significantly higher overall score than the guidelines published by scientific societies or hospitals. Nonetheless, this difference was not substantial across any domain except for the Clarity of presentation. We found no significant differences between the guidelines developed in the last five years or earlier, and between the continents where the CPG was developed. However, the CPGs developed in Asia were better at reporting the aspects related to Applicability, and the most recent CPGs stated Editorial independence more clearly (Table 3).

**Table 3:** Comparison between AGREE II domains and pre-specified predictors

Variables	*n*	Scope and purpose	Stakeholder involvement	Rigour of development	Clarity of presentation	Applicability	Editorial Independence	Overall rate
		Mean (SD)	Mean (SD)	Mean (SD)	Mean (SD)	Mean (SD)	Mean (SD)	Mean (SD)
**Year of development**								
≥ 2018	4	84.0 (14.5)	48.0 (27.5)	50.25 (22.9)	81.3 (12.1)	17.8 (26.5)	64.8 (33.3)	4.75 (1.04)
< 2018	6	72.2 (19.7)	45.3 (32.35)	37.2 (28.6)	72.2 (20.7)	14.5 (14.3)	26.5 (38.3)	3.50 (1.30)
p-value		0.336	0.896	0.468	0.456	0.805	0.1430	0.149
**Type of organization**								
Government	3	91.0 (9.5)	76.0 (9.5)	69.3 (15.5)	71.3 (10.2)	32.0 (28.8)	79.3 (22.3)	5.33 (0.77)
Scientific societies	6	74.3 (16.5)	35.7 (26.2)	33.5 (20.9)	84.7 (9.5)	10.0 (7.8)	28.5 (36.7)	3.67 (0.98)
Hospital	1	50.0 (0.0)	22.0 (0.0)	15.0 (0.0)	36.0 (0.0)	2.0 (0.0)	0.0 (0.0)	2.0 (0.0)
p-value		0.109	0.076	0. 057	0.007*	0.194	0.104	0.027*
**Region**								
Asia	1	92.0 (0.0)	75.0 (0.0)	52.0 (0.0)	64.0 (0.0)	56.0 (0.0)	88.0 (0.0)	4.35 (1.11)
America	2	54.0 (5.7)	12.5 (13.4)	8.5 (9.2)	62.5 (37.5)	1.0 (1.4)	0.0 (0.0)	2.25 (0.35)
Europa	7	81.3 (15.6)	52.0 (25.8)	50.7 (22.8)	81.3 (10.5)	14.3 (13.4)	45.9 (38.8)	5.0 (0.0)
p-value		0.097	0.129	0.106	0.359	0.024*	0.179	0.08

* Statistically significant difference

A significant strong correlation was observed between the scores of the AGREE II domains and the overall rate, excepting the Clarity of presentation (*r =* 0.32; p = 0.363) and Applicability domains (*r* = 0.43; p = 0.21) (Table 4).

**Table 4:** Correlation score between the scores for each AGREE II instrument domain and the overall

Analysis	Scope and purpose	Stakeholder involvement	Rigour of development	Clarity of presentation	Applicability	Editorial Independence
*r*	0.8722	0.7319	0.925	0.342	0.421	0.743
p-value	< 0.001*	0.016*	< 0.001*	0.333	0.226	0.014*

* Statistically significant difference

## Discussion

Our research showed that the overall quality of CPGs in the field of dental trauma is suboptimal; only two out of ten CPGs were assessed as high quality. The domain with the highest score was Scope and Purpose (mean 78.0%), while Applicability obtained the lowest score (mean 15.3%). The AGREE II overall mean rate was 4.0 (SD 1.3). Only two CPGs were recommended without modifications by the reviewers.

The only study variable associated with the quality of the guidelines was the organization responsible for developing the guidelines, since the CPGs developed by governments were found to present the best quality. Finally, as expected, the domain that correlated best with a high-quality CPG was Rigour of development.

Our review showed that the two best-assessed domains were Scope and purpose and Clarity of presentation, consistent with other systematic reviews [30, 31].

Although the Scope and purpose domain passed the quality threshold, some guidelines failed to describe the health questions covered by the CPG. Well formulated study questions help directly the search for evidence, as well as the assessment of certainty; therefore, when choosing which questions to include, the objective and scope of the guide are being defined [32]. Since the recommendations arise from the answers to these questions, the object of the CPG should be clear, and consistent with the recommendations, in order to help the user to implement the most appropriate care for a given patient.

The Clarity of presentation domain presented the second-best evaluation, the main issues being ambiguity and the format in which the recommendations were presented. This is important for making the recommendation easier to implement [18].

As in our study, evidence shows that dental CPGs of different dental specialties tend to be of low quality, presenting important flaws in their development, especially related to Stakeholder involvement, poor Methodological rigour [10, 33, 34], and issues in the Applicability and the Editorial independence domains [12, 13].

Regarding the Stakeholder involvement domain, the views and preferences of the target population were not considered in formulating the recommendations, either because they were not included as members of the panel or because the study did not carry out a systematic search of the evidence. The principal justification for including patients’ values and preferences in guideline development is because recommendations that are in line with these might be more easily accepted, implemented and adhered to by those who will benefit from them [35]. Moreover, most CPGs should have stated the specialists or experts involved in their development. CPGs improve when specialists, methodologists and patients participate actively in guideline development [36, 37].

Rigour of development is regarded as the most important domain for assessing CPGs, since it appraises the process for gathering and synthesizing the evidence, and the methodology for formulating the recommendations. Although most of the CPGs reported carrying out a systematic search of the evidence, only a few assessed the strengths and limitations of the identified evidence. This assessment is critical, since most evidence supporting the recommendations comes from observational or animal studies. The certainty of the evidence in these cases is low or very low, which means there is uncertainty about whether the identified evidence is appropriate to formulate a recommendation (e.g., there is very little evidence or studies have significant limitations). Low or very low certainty of evidence determining a conditional or weak recommendation means that many individuals in this position might accept the suggested course of action, but an important amount would not [38].

Another important limitation in the methodological rigour of the CPGs was that the methodology for formulating the recommendations was not clearly described. Although most of the CPGs reported that the recommendations were formulated by consensus of the panel members, few provided information on the methodology, the factors considered, and the results of the deliberation process. One way to make this process more transparent is through the GRADE approach (Grading of Recommendation Assessment, Development and Evaluation). This methodology provides a structured process for determining the certainty of the evidence, making recommendations, and taking decisions. The GRADE approach does not only consider the quality of the evidence when formulating a recommendation, but also considers the benefit-risk balance, the patients’ values and preferences, the magnitude of the necessary resources and costs, as well as equity, acceptability, and implementation, among others. Evidence shows that the best quality CPGs are those based on evidence and used a transparent way to develop recommendations, like the GRADE methodology [12]. This is important given that poor quality guidelines may negatively influence patient care, or their applicability may be questionable [14, 15]. In our study, only one guideline used the GRADE approach to assess the certainty of evidence and in developing its recommendations, despite the fact that more than 90 health organizations around the world have endorsed this approach [39]. However, this deficiency is also observed in CPGs published for other areas of dentistry [11, 13, 40].

The Applicability domain is poorly reported in CPGs, not only in dental guidelines but also in other health fields [31]. This shows the importance of considering aspects such as implementation, organizational barriers and facilitators, and economic implications when developing future guidelines on TDIs. Inappropriate analysis of these factors can influence adherence to the guideline. When carrying out this analysis, the CPGs must consider the local facilitators and the barriers that may influence their applicability. According to Alonso-Coello et al., low scores in the applicability domain could result from the fact that the developers consider guideline development and guideline implementation as different activities [31].

The Editorial Independence domain was assessed as very low-quality because the CPGs did not declare possible intellectual and financial conflicts of interest. This is a generalized problem, in both dental and medical guidelines [30, 31] [41]. It is essential that both funding bodies and members of CPG development groups state their conflicts in detail, because they are used for decision-making in both insurance coverage and standards of care [42]. It is important to link the recommendations clearly to the evidence, and to exclude panelists with conflicts of interest, in order to avoid influence from external interests [41].

Concerning the factors associated with guideline quality, we observed that guidelines developed by Governments have higher scores than CPGs produced by scientific societies or hospitals, in agreement with the reports of other studies [31, 43]. This is attributable to the large amount of financial and human resources needed to properly develop a CPG [44].

The greatest strength of the present study was that CPGs were obtained by a systematic literature search that included developers’ websites and repositories of CPGs. AGREE II is the only reliable, validated instrument developed for comparing CPGs [18].

### Limitations of the review process

Our study is not exempt from limitations. Although a comprehensive search including gray literature was conducted, relevant guidelines may exist in a language other than those considered in our methodology. Likewise, it is important to note that the recommendations of the CPGs assessed should be viewed cautiously, since AGREE II only appraises the methodological quality of the reporting of CPGs, without judging the rationality of the recommendations made. Other approaches, such as GRADE, should be used to assess the certainty of evidence supporting the recommendations.

### Implications for practice and research

This research highlights the importance of improving the development processes for CPGs in dental traumatology. It is crucial for dentists to identify reliable CPGs before implementing recommendations. Guideline development groups must prioritize quality improvements using a transparent, standardized framework when presenting recommendations. This framework should detail the methodology used, including the method for evaluating the body of evidence and the process by which the guideline panel reaches consensus. In addition, the guideline development process should also consider various aspects, such as the balance between desirable and undesirable effects of interventions, patient values and preferences, certainty of evidence, cost-effectiveness, impact on health equity, stakeholders’ acceptability, and feasibility of implementation.

Future research should be aimed at pursuing strategies to develop evidence-based recommendations when published direct evidence is lacking. Although some initiatives have emerged [45], there is still a lack of research on how to incorporate these methodologies efficiently during the CPG development process where the evidence is scarce or has significant limitations.

Since developing trustworthy guidelines requires substantial time and resources investment, adapting or adopting existing high-quality guidelines is an efficient alternative to developing de novo guidelines.

## Conclusion

The overall quality of CPGs for the diagnosis, emergency management, and follow-up of TDIs was suboptimal, with only two high-quality guidelines out of the ten assessed, making implementation challenging for dentists and policymakers. It is essential that guideline developers should use a methodology that allows them to formulate the recommendations in a structured, transparent, and explicit way.

### Electronic supplementary material

Below is the link to the electronic supplementary material.

Supplementary Material 1

Supplementary Material 2

## Data Availability

The datasets used and/or analyzed during the current study are available from the corresponding author upon reasonable request.

## Abbreviations

AGREE II: Appraisal of Guidelines Research & Evaluation
CPGs: Clinical Practice Guidelines
GRADE: Grading of Recommendation Assessment, Development and Evaluation
TDIs: Traumatic dental injuries
