# Supplementary material for: Emergency and sequalae management of traumatic dental injuries: a systematic survey of clinical practice guidelines
Source: BMC Oral Health. 2023 Sep 30;23:704. doi: 10.1186/s12903-023-03409-w (PMC10543333; doi:10.1186/s12903-023-03409-w)
Supplement: Supplementary file 1 — Supplementary Material 1 [file 12903_2023_3409_MOESM1_ESM.docx]

**APPENDIX S1**

**SEARCH STRATEGY USED IN EACH DATABASE**

| **Source** | **Strategy** | **Results** |
| --- | --- | --- |
| Medline | (((((((((tooth[tiab]) OR teeth) OR dental[tiab]) OR dentoalveolar)) AND (((((injur*[tiab]) OR traumatology[tiab]) OR "Traumatology"[Mesh]) OR ("Wounds and Injuries"[Mesh])) OR trauma[tiab]))))) AND ((((((((((((guideline*[ti]) OR recommendation*[ti]) OR protocol*[ti]) OR consensus[ti]) OR practice guideline[ti]) OR guidance[ti]) OR "Guideline" [Publication Type]) OR "Practice Guideline" [Publication Type]) OR "Health Planning Guidelines"[Mesh]) OR "Consensus"[Mesh]) OR "Guidelines as Topic"[Mesh])) | 470 |
| Embase | (guideline*:ti OR 'guideline'/exp OR 'practice guideline'/exp OR 'consensus development'/exp OR 'consensus'/exp OR recommendation*:ti OR consensus:ti OR protocol*:ti OR 'protocol'/exp) AND ('traumatic dental injur*':ti,ab OR 'dental trauma':ti,ab OR 'dentoalveolar trauma':ti,ab OR 'tooth injur*':ti,ab OR 'teeth injur*':ti,ab) AND [embase]/lim | 43 |
| Epistemonikos | (title:(traumatic dental injur*) OR abstract:(traumatic dental injur*)) OR (title:(dental trauma) OR abstract:(dental trauma)) OR (title:(dentoalveolar trauma) OR abstract:(dentoalveolar trauma)) OR (title:(tooth injur*) OR abstract:(tooth injur*)) OR (title:(teeth injur*) OR abstract:(teeth injur*)) [Filters: protocol=no, classification=broad-synthesis] | 4 |
| TripDatabase | “traumatic dental injur*” OR “dental trauma” OR “dentoalveolar trauma” OR “tooth injur*” OR “teeth injur*” | 17 |

**WEBSITES**

***Guidelines developers***

- National Institute for Health and Care Excellence (http://www.nice.org.uk)
- Scottish Intercollegiate Guidelines Network (http://www.sign.ac.uk)
- Guía Salud (https://portal.guiasalud.es)
- CMA Infobase: Clinical Practice Guidelines Database (CPGs) (https://joulecma.ca/cpg/homepage)
- Australian Clinical Practice Guideline (https://www.clinicalguidelines.gov.au)
- New Zealand Guidelines (https://www.health.govt.nz/about-ministry/ministry-

health-websites/new-zealand-guidelines-group)

- Scottish Dental Clinical Effectiveness Programme (http://www.sdcep.org.uk)
- EBM Guidelines (https://www.ebm-guidelines.com/dtk/ebmg/home)
- IECS: Instituto de Efectividad Clínica y Sanitaria (https://www.iecs.org.ar).

***CPGs compiler entities***

- National Guideline Clearinghouse (http://www.guideline.gov)
- Guideline International Networks (GIN): (http://www.g-i-n.net/library/international-guidelines-library)
- ECRI Guidelines Trust (https://guidelines.ecri.org/)
- NeLH Guidelines Finder (http://libraries.nelh.nhs.uk/guidelinesFinder/)
- Agency for Healthcare Research and Quality (AHRQ)

https://www.ahrq.gov/research/findings/evidence-based-reports/search.html)

- Guideline Central(https://www.guidelinecentral.com/)
- The Alliance for the Implementation of Clinical Practice Guidelines

(https://aicpg.org/)

- Scottish dental (https://www.scottishdental.org/professionals/guidelines/)

***Scientific Societies and Health Organizations***

- World health Organization (OMS) (https://www.who.int/es)
- Organización Panamericana de la Salud (OPS) (https://www.paho.org/en)
- American Dental Association (ADA) (https://www.ada.org/en)
- FDI World Dental Association (https://www.fdiworlddental.org)
- International Association for Dental Research (IADR)(https://www.iadr.org)
- American Academy of Pediatric Dentistry (AAPD) (http://www.aapd.org/)
- Australian and New Zealand Society of Pediatric Dentistry (ANZSPD)

(https://www.anzspd.org.au/)

- European Academy of Paediatric Dentistry (EAPD) (http://www.eapd.gr/)
- International Association of Paediatric Dentistry (IAPD)

(http://www.iapdworld.org/)

- Pediatric Dentistry Association of Asia (PDAA) (<http://pdaasia.org/>)
- International association of dental traumatology (<https://www.iadt-dentaltrauma.org>)
- International association of oral and maxillofacial surgeons (<https://www.iaoms.org>)
- International Association of DentoMaxilloFacial Radiology (IADMFR) (<https://iadmfr.one>)
- International Federation of Endodontic Associations (http://www.ifeaendo.org/about-us/what-is-ifea/)
- American association of endodontists (<https://www.aae.org>)
- European society of endodontology (<https://www.e-s-e.eu>)

***Ministries of Health***

- Africa: Nigeria (http://www.fmh.gov.ng), South Africa (http://www.doh.gov.za/), Sudan (http://www.fmoh.gov.sd/), Tanzania (http://www.moh.go.tz/) and Uganda (http://health.go.ug/mohweb/).
- America: Argentina (https://www.argentina.gob.ar/salud), Brasil (https://saude.gov.br), Bolivia (https://www.minsalud.gob.bo), Chile (http://www.minsal.cl), Colombia (https://www.minsalud.gov.co/portada-covid- 19.html), Costa Rica (http://www.ministeriodesalud.go.cr/), Cuba (http://www.sld.cu/), Dominican Republic (http://www.salud.gob.do/), El Salvador (http://www.salud.gob.sv/), Nicaragua and (http://www.minsa.gob.ni/), Perú (https://www.gob.pe/minsa/), Trinidad and Tobago (http://www.health.gov.tt/) and United States of America (http://www.hhs.gov/).
- Asia: Bhutan (http://www.health.gov.bt/), Cambodia (http://www.moh.gov.kh/), China (http://www.moh.gov.cn), Indonesia (http://www.depkes.go.id), Iraq (http://www.moh.gov.iq),
  Japon (<http://www.mhlw.go.jp/>), Pakistan (www.pakistan.gov.pk), Singapore (http://www.health.gov.lk/), Thailand (http://eng.moph.go.th/) and Turkey (http://www.sb.gov.tr/).
- Europe: Austria (http://www.bmg.gv.at/), Belgium (http://www.health.belgium.be), Cyprus (http://www.moh.gov.cy), Denmark (http://www.sst.dk/), England (http://www.dh.gov.uk), France (http://www.sante.gouv.fr/), Germany (http://www.bmg.bund.de), Greece (http://www.yyka.gov.gr/), Iceland (http://www.velferdarraduneyti.is/), Ireland (http://www.dohc.ie), Italy (http://www.salute.gov.it/), Netherlands (http://www.government.nl/ministries/vws), Norway (http://www.regjeringen.no), Portugal (http://www.portaldasaude.pt), Spain (http://www.msc.es/), Sweden (http://www.folktandvardenstockholm.se/) and Switzerland (http://www.bag.admin.ch).
- Oceania: Australia (http://www.health.gov.au/) and New Zealand (http://www.health.govt.nz).
